# Semisynthetic roxburghin tetra­methyl ether

**DOI:** 10.1107/S1600536808018266

**Published:** 2008-06-19

**Authors:** Alex Saez, Carmen Ramirez de Arellano, Noureddine El Aouad, Silvia Rodriguez, Felipe Otalvaro, Diego Cortes, Jairo Saez

**Affiliations:** a Instituto de Química, Química de Plantas Colombianas, Universidad de Antioquia, AA 1226, Medellín, Colombia; b Grupo de Procesos Ambientales y Biotecnológicos, Departamento de Ingeniería de Procesos, Universidad EAFIT, AA 3300, Medellín, Colombia; c Departamento de Química Orgánica, Universidad de Valencia, E-46100 Valencia, Spain; d Departamento de Farmacología, Facultad de Farmacia, Universidad de Valencia, Burjassot, Valencia, Spain

## Abstract

The title mol­ecule, (*E*)-2,3′,4,5-tetra­methoxy­stilbene, C_18_H_20_O_4_, is virtually planar. The angle between the two benzene rings is 4.06 (6)°. The inter­molecular inter­actions present in the structure are weak. There are C—H⋯O hydrogen bonds and C—H⋯π-electron ring inter­actions. The mol­ecules are ordered into planes that are parallel to (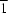
01). The distance between adjacent planes is about 3.3 Å and therefore π–π electron inter­actions between the aromatic planes are also plausible.

## Related literature

For the importance and useful applications of stilbenoid compounds, see: Cushman *et al.* (1991 ▶); Nakamura *et al.* (2006 ▶). For the precursors of the title compound, see: Krishnamurty & Maheshwari (1988 ▶); Anjaneyulu *et al.* (1990 ▶); Wang *et al.* (1988 ▶); Murillo (2001 ▶).
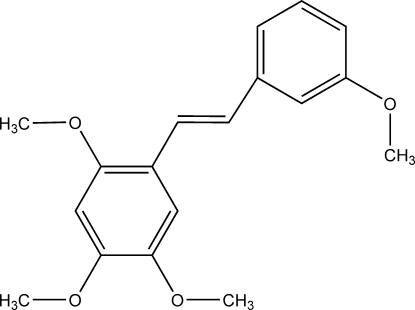


## Experimental

### 

#### Crystal data

C_18_H_20_O_4_
*M* *_r_* = 300.34
Triclinic, 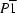

*a* = 7.9633 (4) Å
*b* = 9.2454 (5) Å
*c* = 11.6194 (5) Å
α = 73.400 (2)°
β = 75.479 (3)°
γ = 70.335 (2)°
*V* = 760.59 (7) Å^3^
*Z* = 2
Mo *K*α radiation
μ = 0.09 mm^−1^
*T* = 150 (2) K
0.35 × 0.10 × 0.04 mm

#### Data collection

Nonius KappaCCD diffractometer
Absorption correction: none
8260 measured reflections
4391 independent reflections
2785 reflections with *I* > 2σ(*I*)
*R*_int_ = 0.025

#### Refinement

*R*[*F* ^2^ > 2σ(*F* ^2^)] = 0.047
*wR*(*F* ^2^) = 0.136
*S* = 0.98
4391 reflections
203 parameters
H-atom parameters constrained
Δρ_max_ = 0.29 e Å^−3^
Δρ_min_ = −0.23 e Å^−3^

### 

Data collection: *COLLECT* (Nonius, 1998 ▶); cell refinement: *SCALEPACK* (Otwinowski & Minor, 1997 ▶); data reduction: *SCALEPACK*; program(s) used to solve structure: *SHELXS97* (Sheldrick, 2008 ▶); program(s) used to refine structure: *SHELXL97* (Sheldrick, 2008 ▶); molecular graphics: *SHELXTL/PC* (Sheldrick, 2008 ▶); software used to prepare material for publication: *SHELXL97*.

## Supplementary Material

Crystal structure: contains datablocks global, I. DOI: 10.1107/S1600536808018266/fb2091sup1.cif

Structure factors: contains datablocks I. DOI: 10.1107/S1600536808018266/fb2091Isup2.hkl

Additional supplementary materials:  crystallographic information; 3D view; checkCIF report

## Figures and Tables

**Table 1:** Hydrogen-bond geometry (Å, °)

*D*—H⋯*A*	*D*—H	H⋯*A*	*D*⋯*A*	*D*—H⋯*A*
C7—H7⋯O3	0.95	2.39	2.7504 (14)	102
C17—H17*B*⋯O3^i^	0.98	2.52	3.4046 (16)	150
C18—H18*B*⋯O4^ii^	0.98	2.46	3.4342 (15)	172
C19—H19*B*⋯O1^iii^	0.98	2.51	3.4082 (15)	152
C17—H17*A*⋯*Cg*2^iv^	0.98	2.91	3.7863 (15)	149
C18—H18*C*⋯*Cg*1^v^	0.98	2.67	3.5578 (14)	151

Symmetry codes: (i) 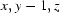; (ii) 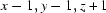; (iii) 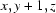; (iv) 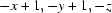; (v) 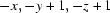. *Cg*1 is the centroid of the C1–C6 ring and Cg2 is the centroid of the C11–C16 ring.

**Fig. 1.**
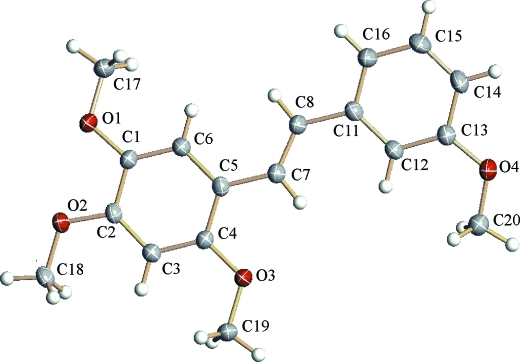
The title molecule with the displacement ellipsoids shown at the 50% probability level.

## supplementary crystallographic information

### Comment

Stilbenoid compounds display significant biological activities (Cushman *et al.*, 1991; Nakamura *et al.*, 2006). Resveratrol and its derivatives deserve considerable attention for their physiological properties and their role in defense mechanisms of the higher plants. The Roxburghin tetramethyl ether (*E*)-2,3',4,5,-tetramethoxystilbene) that is an analogue of resveratrol, has been originally obtained by modifications of roxburghin (Krishnamurty & Maheshwari, 1988). It has been completely synthesized by the Perkins modified reaction (Anjaneyulu *et al.*, 1990). In addition to the crystal structure determination, we report an efficient synthesis of this product by the cross-metathesis of 3-methoxystyrene and 2,4,5-trimethoxystyrene, the latter having been obtained as a natural product from the bark of *Duguetia colombiana *(Annonaceae) (Wang *et al.*, 1988; Murillo, 2001).

### Experimental

The catalyst (Grubbs second generation, 9 mg, 0.01 mmol), 2,4,5-trimethoxystyrene (39 mg, 0.2 mmol) and 2-methoxystyrene (277 mg, 2.0 mmol) were disolved in dry toluene (10 ml). The solution was refluxed under nitrogen for 24 h at 393 K. The compound was purified by a flash column chromatography with silica gel using hexane/ethylacetate 9:1 as an eluent. The title compound (30.0 mg) was obtained as a yellow powder in a yield of 50.0%.

Suitable crystals (pale yellow needles, 0.35 x 0.10 x 0.04 mm average size) were obtained by slow evaporation in a two solvent system (hexane/ethylacetate 1:1). The identity and purity of the obtained compound was confirmed by spectroscopic methods.

(*E*)-1,2,4-trimethoxy-5-(3-methoxystyryl)benzene(Roxburghin tetramethyl ether): pale yellow needles, ^1^H-NMR: (CDCl_3_,300.13 MHz, numeration acording to ellipsoid plot) d 7.42 (d, *J*= 16.4 Hz, H-7), 7.26 (dd, *J* = 8.3,7.7 Hz, H-15), 7.12 (s, H-6), 7.12 (d, *J*= 7.7 Hz, H-16), 7.06 (s, H-12), 6.79 (d, *J*= 8.3 Hz, H-14), 6.54 (s, H-3), 3.92 (s, C-2-OCH3),3.92 (s, C-1-OCH3), 3.87 (s, C-4-OCH3),3.85 (s, C-13-OCH3); ^13^C (CDCl_3_, 75.47 MHz) d 160.2 (C-13), 152.2 (C-4), 150.1 (C-2),143.8 (C-1), 140.0 (C-11), 129.9 (C-15), 127.1 (C-8), 123.7 (C-7), 119.5 (C-16),118.6 (C-5), 113.1 (C-12), 111.9 (C-6), 109.8 (C-14), 98.1 (C-3), 57.1 (C-17), 56.9(C-18), 56.5 (C-19), 55.7 (C-20). EIMS *m/z*300 (100), 257 (8), 195 (12).

### Refinement

All the H atoms were discernible in the difference electron-density maps. However, they were situated into idealized positions and constrained by riding model approximation. *C*—H_methyl_=0.98 Å; *C*—H_aryl_=0.95 Å; *U*_iso_H_methyl_=1.5*U*_eq_(C_methyl_); *U*_iso_H_aryl_=1.2*U*_eq_(C_aryl_).

### Crystal data

**Table:**

C_18_H_20_O_4_	*Z* = 2
*M**_r_* = 300.34	*F*_000_ = 320
Triclinic, *P*1	*D*_x_ = 1.311 Mg m^−^^3^
Hall symbol: -P 1	Mo *K*α radiation λ = 0.71073 Å
*a* = 7.9633 (4) Å	Cell parameters from 46078 reflections
*b* = 9.2454 (5) Å	θ = 1.0–30.0º
*c* = 11.6194 (5) Å	µ = 0.09 mm^−^^1^
α = 73.400 (2)º	*T* = 150 (2) K
β = 75.479 (3)º	Needle, yellow
γ = 70.335 (2)º	0.35 × 0.10 × 0.04 mm
*V* = 760.59 (7) Å^3^	

### Data collection

**Table:**

Nonius KappaCCD diffractometer	4391 independent reflections
Radiation source: fine-focus sealed tube	2785 reflections with *I* > 2σ(*I*)
Monochromator: graphite	*R*_int_ = 0.025
Detector resolution: 9 pixels mm^-1^	θ_max_ = 30.0º
*T* = 150(2) K	θ_min_ = 2.7º
ω scans	*h* = −11→11
Absorption correction: none	*k* = −13→12
8260 measured reflections	*l* = −16→16

### Refinement

**Table:**

Refinement on *F*^2^	Secondary atom site location: difference Fourier map
Least-squares matrix: full	Hydrogen site location: difference Fourier map
*R*[*F*^2^ > 2σ(*F*^2^)] = 0.047	H-atom parameters constrained
*wR*(*F*^2^) = 0.136	*w* = 1/[σ^2^(*F*_o_^2^) + (0.0789*P*)^2^] where *P* = (*F*_o_^2^ + 2*F*_c_^2^)/3
*S* = 0.98	(Δ/σ)_max_ < 0.001
4391 reflections	Δρ_max_ = 0.29 e Å^−^^3^
203 parameters	Δρ_min_ = −0.23 e Å^−^^3^
76 constraints	Extinction correction: none
Primary atom site location: structure-invariant direct methods	

### Special details

**Table:**

Geometry. All e.s.d.'s (except the e.s.d. in the dihedral angle between two l.s. planes) are estimated using the full covariance matrix. The cell e.s.d.'s are taken into account individually in the estimation of e.s.d.'s in distances, angles and torsion angles; correlations between e.s.d.'s in cell parameters are only used when they are defined by crystal symmetry. An approximate (isotropic) treatment of cell e.s.d.'s is used for estimating e.s.d.'s involving l.s. planes.
Refinement. Refinement of *F*^2^ against ALL reflections. The weighted *R*-factor *wR* and goodness of fit *S* are based on *F*^2^, conventional *R*-factors *R* are based on *F*, with *F* set to zero for negative *F*^2^. The threshold expression of *F*^2^ > σ(*F*^2^) is used only for calculating *R*-factors(gt) *etc*. and is not relevant to the choice of reflections for refinement. *R*-factors based on *F*^2^ are statistically about twice as large as those based on *F*, and *R*- factors based on ALL data will be even larger.

### Fractional atomic coordinates and isotropic or equivalent isotropic displacement parameters (Å^2^)

**Table:**

	*x*	*y*	*z*	*U*_iso_*/*U*_eq_	
O1	0.35419 (11)	0.13391 (9)	0.41479 (8)	0.0342 (2)	
O2	0.16009 (11)	0.25274 (9)	0.59974 (7)	0.0302 (2)	
O3	0.31164 (11)	0.74440 (9)	0.41175 (7)	0.0322 (2)	
O4	0.83171 (12)	1.06354 (9)	−0.08834 (8)	0.0352 (2)	
C1	0.34968 (15)	0.28542 (12)	0.40914 (10)	0.0257 (2)	
C2	0.24495 (14)	0.34919 (12)	0.50941 (9)	0.0242 (2)	
C3	0.23241 (14)	0.50121 (12)	0.51169 (10)	0.0252 (2)	
H3	0.1620	0.5441	0.5796	0.030*	
C4	0.32276 (14)	0.59219 (12)	0.41457 (10)	0.0242 (2)	
C5	0.42856 (14)	0.53130 (12)	0.31388 (10)	0.0237 (2)	
C6	0.43875 (15)	0.37648 (12)	0.31392 (10)	0.0260 (2)	
H6	0.5092	0.3330	0.2463	0.031*	
C7	0.52275 (15)	0.62808 (13)	0.21313 (10)	0.0255 (2)	
H7	0.5008	0.7340	0.2176	0.031*	
C8	0.63618 (15)	0.58230 (13)	0.11577 (10)	0.0286 (2)	
H8	0.6583	0.4762	0.1116	0.034*	
C11	0.73066 (15)	0.67918 (13)	0.01411 (10)	0.0266 (2)	
C12	0.72987 (14)	0.83038 (13)	0.01567 (10)	0.0257 (2)	
H12	0.6661	0.8741	0.0844	0.031*	
C13	0.82170 (15)	0.91678 (13)	−0.08266 (10)	0.0275 (3)	
C14	0.91383 (17)	0.85479 (15)	−0.18482 (11)	0.0352 (3)	
H14	0.9754	0.9144	−0.2524	0.042*	
C15	0.91459 (18)	0.70674 (15)	−0.18674 (11)	0.0400 (3)	
H15	0.9770	0.6642	−0.2562	0.048*	
C16	0.82497 (17)	0.61818 (14)	−0.08813 (11)	0.0348 (3)	
H16	0.8281	0.5154	−0.0906	0.042*	
C17	0.48206 (17)	0.05869 (13)	0.32371 (12)	0.0372 (3)	
H17A	0.4483	0.1127	0.2435	0.056*	
H17B	0.4825	−0.0516	0.3411	0.056*	
H17C	0.6030	0.0634	0.3241	0.056*	
C18	0.06092 (16)	0.31142 (13)	0.70597 (10)	0.0303 (3)	
H18A	0.1428	0.3348	0.7439	0.046*	
H18B	0.0074	0.2319	0.7640	0.046*	
H18C	−0.0353	0.4077	0.6828	0.046*	
C19	0.19201 (16)	0.81424 (13)	0.50742 (11)	0.0329 (3)	
H19A	0.0689	0.8124	0.5097	0.049*	
H19B	0.1939	0.9235	0.4930	0.049*	
H19C	0.2309	0.7550	0.5854	0.049*	
C20	0.75180 (17)	1.12894 (14)	0.01691 (11)	0.0349 (3)	
H20A	0.6208	1.1449	0.0326	0.052*	
H20B	0.7762	1.2302	0.0029	0.052*	
H20C	0.8036	1.0566	0.0875	0.052*	

### Atomic displacement parameters (Å^2^)

**Table:**

	*U*^11^	*U*^22^	*U*^33^	*U*^12^	*U*^13^	*U*^23^
O1	0.0446 (5)	0.0235 (4)	0.0337 (5)	−0.0156 (3)	0.0077 (4)	−0.0104 (3)
O2	0.0344 (4)	0.0262 (4)	0.0267 (4)	−0.0133 (3)	0.0047 (3)	−0.0040 (3)
O3	0.0395 (5)	0.0247 (4)	0.0317 (4)	−0.0148 (3)	0.0080 (4)	−0.0108 (3)
O4	0.0432 (5)	0.0296 (4)	0.0328 (5)	−0.0187 (4)	0.0041 (4)	−0.0064 (4)
C1	0.0290 (6)	0.0202 (5)	0.0279 (6)	−0.0089 (4)	−0.0023 (5)	−0.0054 (4)
C2	0.0240 (5)	0.0245 (5)	0.0221 (5)	−0.0092 (4)	−0.0019 (4)	−0.0015 (4)
C3	0.0251 (5)	0.0255 (5)	0.0243 (5)	−0.0078 (4)	−0.0008 (4)	−0.0066 (4)
C4	0.0256 (5)	0.0212 (5)	0.0264 (6)	−0.0083 (4)	−0.0024 (4)	−0.0062 (4)
C5	0.0231 (5)	0.0239 (5)	0.0242 (5)	−0.0089 (4)	−0.0024 (4)	−0.0040 (4)
C6	0.0278 (6)	0.0255 (5)	0.0244 (5)	−0.0095 (4)	0.0002 (4)	−0.0069 (4)
C7	0.0272 (6)	0.0238 (5)	0.0255 (6)	−0.0097 (4)	−0.0024 (5)	−0.0045 (4)
C8	0.0339 (6)	0.0233 (5)	0.0279 (6)	−0.0116 (4)	0.0006 (5)	−0.0056 (4)
C11	0.0261 (5)	0.0276 (6)	0.0244 (5)	−0.0091 (4)	−0.0007 (4)	−0.0046 (4)
C12	0.0259 (5)	0.0282 (5)	0.0217 (5)	−0.0089 (4)	0.0002 (4)	−0.0059 (4)
C13	0.0274 (6)	0.0281 (6)	0.0266 (6)	−0.0105 (5)	−0.0020 (5)	−0.0048 (5)
C14	0.0397 (7)	0.0398 (7)	0.0259 (6)	−0.0210 (6)	0.0055 (5)	−0.0051 (5)
C15	0.0488 (8)	0.0436 (7)	0.0278 (6)	−0.0192 (6)	0.0094 (6)	−0.0149 (6)
C16	0.0434 (7)	0.0302 (6)	0.0309 (6)	−0.0158 (5)	0.0058 (5)	−0.0112 (5)
C17	0.0439 (7)	0.0279 (6)	0.0381 (7)	−0.0126 (5)	0.0071 (6)	−0.0141 (5)
C18	0.0343 (6)	0.0345 (6)	0.0211 (5)	−0.0152 (5)	0.0019 (5)	−0.0038 (5)
C19	0.0353 (6)	0.0276 (6)	0.0358 (7)	−0.0111 (5)	0.0055 (5)	−0.0142 (5)
C20	0.0374 (7)	0.0310 (6)	0.0373 (7)	−0.0134 (5)	0.0003 (5)	−0.0104 (5)

### Geometric parameters (Å, °)

**Table:**

O1—C1	1.3720 (12)	C11—C12	1.4011 (15)
O1—C17	1.4298 (13)	C12—C13	1.3896 (15)
O2—C2	1.3670 (13)	C12—H12	0.9500
O2—C18	1.4308 (13)	C13—C14	1.3945 (16)
O3—C4	1.3713 (12)	C14—C15	1.3733 (16)
O3—C19	1.4231 (13)	C14—H14	0.9500
O4—C13	1.3677 (13)	C15—C16	1.3927 (17)
O4—C20	1.4281 (13)	C15—H15	0.9500
C1—C6	1.3828 (15)	C16—H16	0.9500
C1—C2	1.4052 (14)	C17—H17A	0.9800
C2—C3	1.3826 (14)	C17—H17B	0.9800
C3—C4	1.3988 (15)	C17—H17C	0.9800
C3—H3	0.9500	C18—H18A	0.9800
C4—C5	1.3994 (14)	C18—H18B	0.9800
C5—C6	1.4060 (14)	C18—H18C	0.9800
C5—C7	1.4651 (15)	C19—H19A	0.9800
C6—H6	0.9500	C19—H19B	0.9800
C7—C8	1.3336 (16)	C19—H19C	0.9800
C7—H7	0.9500	C20—H20A	0.9800
C8—C11	1.4721 (15)	C20—H20B	0.9800
C8—H8	0.9500	C20—H20C	0.9800
C11—C16	1.3942 (15)		
			
C1—O1—C17	116.35 (8)	O4—C13—C14	115.01 (9)
C2—O2—C18	117.08 (8)	C12—C13—C14	120.36 (10)
C4—O3—C19	117.81 (8)	C15—C14—C13	119.33 (10)
C13—O4—C20	117.84 (8)	C15—C14—H14	120.3
O1—C1—C6	125.08 (10)	C13—C14—H14	120.3
O1—C1—C2	115.75 (9)	C14—C15—C16	120.85 (11)
C6—C1—C2	119.18 (9)	C14—C15—H15	119.6
O2—C2—C3	124.25 (10)	C16—C15—H15	119.6
O2—C2—C1	115.93 (9)	C15—C16—C11	120.49 (10)
C3—C2—C1	119.82 (9)	C15—C16—H16	119.8
C2—C3—C4	120.40 (10)	C11—C16—H16	119.8
C2—C3—H3	119.8	O1—C17—H17A	109.5
C4—C3—H3	119.8	O1—C17—H17B	109.5
O3—C4—C3	122.49 (9)	H17A—C17—H17B	109.5
O3—C4—C5	116.58 (9)	O1—C17—H17C	109.5
C3—C4—C5	120.93 (9)	H17A—C17—H17C	109.5
C4—C5—C6	117.45 (9)	H17B—C17—H17C	109.5
C4—C5—C7	120.20 (9)	O2—C18—H18A	109.5
C6—C5—C7	122.35 (10)	O2—C18—H18B	109.5
C1—C6—C5	122.22 (10)	H18A—C18—H18B	109.5
C1—C6—H6	118.9	O2—C18—H18C	109.5
C5—C6—H6	118.9	H18A—C18—H18C	109.5
C8—C7—C5	126.52 (10)	H18B—C18—H18C	109.5
C8—C7—H7	116.7	O3—C19—H19A	109.5
C5—C7—H7	116.7	O3—C19—H19B	109.5
C7—C8—C11	126.74 (10)	H19A—C19—H19B	109.5
C7—C8—H8	116.6	O3—C19—H19C	109.5
C11—C8—H8	116.6	H19A—C19—H19C	109.5
C16—C11—C12	118.51 (10)	H19B—C19—H19C	109.5
C16—C11—C8	118.73 (10)	O4—C20—H20A	109.5
C12—C11—C8	122.76 (10)	O4—C20—H20B	109.5
C13—C12—C11	120.45 (10)	H20A—C20—H20B	109.5
C13—C12—H12	119.8	O4—C20—H20C	109.5
C11—C12—H12	119.8	H20A—C20—H20C	109.5
O4—C13—C12	124.63 (10)	H20B—C20—H20C	109.5

### Hydrogen-bond geometry (Å, °)

**Table:**

*D*—H···*A*	*D*—H	H···*A*	*D*···*A*	*D*—H···*A*
C7—H7···O3	0.95	2.39	2.7504 (14)	102
C17—H17B···O3^i^	0.98	2.52	3.4046 (16)	150
C18—H18B···O4^ii^	0.98	2.46	3.4342 (15)	172
C19—H19B···O1^iii^	0.98	2.51	3.4082 (15)	152
C17—H17A···Cg2^iv^	0.98	2.91	3.7863 (15)	149
C18—H18C···Cg1^v^	0.98	2.67	3.5578 (14)	151

Symmetry codes: (i) *x*, *y*−1, *z*; (ii) *x*−1, *y*−1, *z*+1; (iii) *x*, *y*+1, *z*; (iv) −*x*+1, −*y*+1, −*z*; (v) −*x*, −*y*+1, −*z*+1.
